# An overview of the *Phalaenopsis *orchid genome through BAC end sequence analysis

**DOI:** 10.1186/1471-2229-11-3

**Published:** 2011-01-06

**Authors:** Chia-Chi Hsu, Yu-Lin Chung, Tien-Chih Chen, Yu-Ling Lee, Yi-Tzu Kuo, Wen-Chieh Tsai, Yu-Yun Hsiao, Yun-Wen Chen, Wen-Luan Wu, Hong-Hwa Chen

**Affiliations:** 1 Department of Life Sciences, National Cheng Kung University, Tainan 701, Taiwan; 2 Institute of Tropical Plant Sciences, National Cheng Kung University, Tainan 701, Taiwan; 3 Orchid Research Center, National Cheng Kung University, Tainan 701, Taiwan

## Abstract

**Background:**

*Phalaenopsis *orchids are popular floral crops, and development of new cultivars is economically important to floricultural industries worldwide. Analysis of orchid genes could facilitate orchid improvement. Bacterial artificial chromosome (BAC) end sequences (BESs) can provide the first glimpses into the sequence composition of a novel genome and can yield molecular markers for use in genetic mapping and breeding.

**Results:**

We used two BAC libraries (constructed using the *Bam*HI and *Hin*dIII restriction enzymes) of *Phalaenopsis equestris *to generate pair-end sequences from 2,920 BAC clones (71.4% and 28.6% from the *Bam*HI and *Hin*dIII libraries, respectively), at a success rate of 95.7%. A total of 5,535 BESs were generated, representing 4.5 Mb, or about 0.3% of the *Phalaenopsis *genome. The trimmed sequences ranged from 123 to 1,397 base pairs (bp) in size, with an average edited read length of 821 bp. When these BESs were subjected to sequence homology searches, it was found that 641 (11.6%) were predicted to represent protein-encoding regions, whereas 1,272 (23.0%) contained repetitive DNA. Most of the repetitive DNA sequences were gypsy- and copia-like retrotransposons (41.9% and 12.8%, respectively), whereas only 10.8% were DNA transposons. Further, 950 potential simple sequence repeats (SSRs) were discovered. Dinucleotides were the most abundant repeat motifs; AT/TA dimer repeats were the most frequent SSRs, representing 253 (26.6%) of all identified SSRs. Microsynteny analysis revealed that more BESs mapped to the whole-genome sequences of poplar than to those of grape or *Arabidopsis*, and even fewer mapped to the rice genome. This work will facilitate analysis of the *Phalaenopsis *genome, and will help clarify similarities and differences in genome composition between orchids and other plant species.

**Conclusion:**

Using BES analysis, we obtained an overview of the *Phalaenopsis *genome in terms of gene abundance, the presence of repetitive DNA and SSR markers, and the extent of microsynteny with other plant species. This work provides a basis for future physical mapping of the *Phalaenopsis *genome and advances our knowledge thereof.

## Background

The family Orchidaceae, which contains at least 25,000 species, is one of the largest families of flowering plants [1]. As with all other living organisms, present-day orchids have evolved from ancestral forms as a result of selection pressure and adaptation. Orchids show a wide diversity of epiphytic and terrestrial growth forms, and these plants have successfully colonized almost every habitat on earth. The factors promoting the richness of orchid species may include specific interactions between orchid flowers and pollinators [2], sequential and rapid interplay between drift and natural selection [3], obligate orchid-mycorrhizal interactions [4], and epiphytism. The latter mode is the growth form of more than 70% of all orchids [5], which comprise approximately two-thirds of the epiphytic flora of the world.

Expansion of diversity may have taken place more quickly in the orchid family than in most other flowering plant families, which had already started to diversify in the mid-Cretaceous [6]. The time at which orchids originated is disputed, but it has been suggested to be 80-40 million years ago (Mya) (thus in the late Cretaceous to late Eocene) [7]. Recently, the Orchidaceae were dated using an amber fossil of an orchid pollinia on the back of the pollinator, a stingless bee [8]. The most recent common ancestor of extant orchids is believed to have lived in the late Cretaceous (76-84 Mya) [8]. Perhaps the only general statement that can be made about the origin of orchids is that most extant groups are probably very young.

Orchids are known for the diversity of their specialized reproductive and ecological strategies. Formation of the labellum and gynostemium (a fused structure of the androecium and gynoecium) to facilitate pollination has been thoroughly documented, and the co-evolution of orchid flowers and pollinators thereof is well understood [9,10]. The successful evolutionary progress of orchids may be explained by the packaging of mature pollen grains as pollinia, the pollination-based regulation of ovary/ovule development, the synchronized timing of micro- and mega-gametogenesis for effective fertilization, and the release of thousands or millions of immature embryos (endosperm-free seeds) in a mature capsule [11]. However, despite the unique aspects of developmental reproductive biology and the specialized pollination and ecological strategies of orchids, relatively few molecular studies have focused on orchids compared to other species-rich plant families [12].

The genomic sequence resources for orchids are limited. A number of studies have used Sanger sequencing to develop expressed sequence tag (EST) resources for orchids [13-15]. These works have highlighted the usefulness of cDNA sequencing in the discovery of candidate genes for orchid floral development [16,17], floral scent production [14,18], and flowering time determination [19], in the absence of a full genomic sequence. However, we do not yet have a comprehensive description of all genes that are expressed in orchids.

Hybrids of the genus *Phalaenopsis *are among the top-traded blooming potted plants worldwide. Because the plants possess favorable commercial traits, such as numerous spikes and branches, along with many colorful flowers, *P. equestris *is often used as a parent for breeding in its native Taiwan. *P. equestris *is a diploid plant with 38 chromosomes (2n = 2x) that are small and uniform in size (< 2 μm long) [20]. The plant has an estimated haploid genome size of 1,600 Mb (3.37 pg/diploid genome), which is relatively small compared to those of other members of the genus *Phalaenopsis *[21]. Public databases of floral bud ESTs from *P. equestris *and *P. bellina *have been developed and analyzed [14,17]; they provide valuable opportunities for researchers to directly access genes of interest [16-18] and to identify molecular markers useful in marker-assisted breeding programs or cultivar identification (unpublished data). However, we still lack basic information on the sequence, organization, and structure of the *Phalaenopsis *genome.

One efficient and viable strategy for gaining insight into the sequence content and complexity of the *Phalaenopsis *genome is afforded by the construction of bacterial artificial chromosome (BAC) libraries and end-sequencing of randomly selected BAC clones. Such BAC end sequences (BESs) can be used as a primary scaffold for genome shotgun-sequence assembly and to generate comparative physical maps [22]. Analysis of BES data can provide an overview of the sequence composition of a novel genome, yielding information on gene density, and the presence of potential transposable elements (TEs) and microsatellites [23-26]. In addition, BESs can identify molecular markers that may be used for genomic mapping and cloning, and in phylogenetic analysis. Even for the rice genome, which has been fully sequenced, the *Oryza *Map Alignment Project (OMAP) constructed deep-coverage large-insert BAC libraries from 11 wild and 1 cultivated African *Oryza *species (*O. glaberrima*); clones from these 12 BAC libraries were next fingerprinted and end-sequenced. The resulting data were used to construct physical maps of the *Oryza *species to permit studies on evolution, genome organization, domestication, gene regulatory networks, and efforts toward crop improvement [27,28]. However, such work has not yet been performed in orchids.

In the present study, we analyzed 5,535 BESs of two genomic BAC libraries of *P. equestris*, focusing on simple sequence repeat (SSR) or microsatellite content, repeat element composition, GC content, and protein-encoding regions. The annotated BESs reported herein offer the first detailed insights into the sequence composition of the *P. equestris *genome, and should be a useful resource for future molecular marker development.

## Results and Discussion

### BAC end sequencing

Two large-insert bacterial artificial chromosome (BAC) libraries were used for end-sequencing in the present study. One library, constructed from a partial *Hin*dIII digest of *P. equestris *genomic DNA, consisted of 100,992 clones with an average insert size of 100 kb. The other library, constructed from a partial *Bam*HI digest, consisted of 33,428 clones with an average insert size of 111 kb. The two libraries represent approximately 8.4 equivalents of the wild-type *Phalaenopsis *haploid genome.

**Figure 1:**
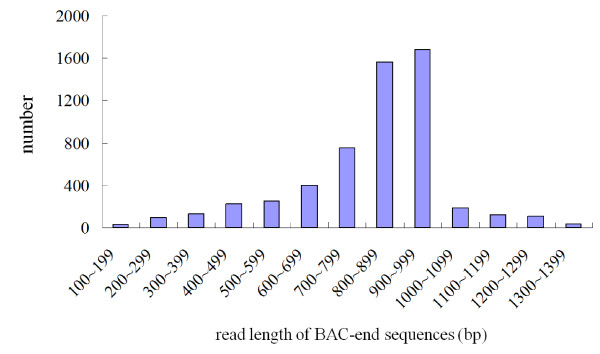
**Size distribution of *Phalaenopsis equestris *bacterial artificial chromosome (BAC) end sequences**. The trimmed sequences ranged from 123 to 1,397 bp in length and had an average edited read length of 821 bp.

DNA samples extracted from 2,920 BAC clones (71.4% and 28.6% from the *Bam*HI and *Hin*dIII libraries, respectively) were sequenced from both ends using Applied Biosystems (ABI) Big Dye terminator chemistry followed by analysis on ABI 3730 machines. The success rate was 95.7%. After ambiguous, vector, and mitochondrial DNA sequences were omitted, 5,535 high-quality BESs remained; these included 5,360 paired-end reads (Table 1). The BESs ranged in size from 123 to 1,397 bp (average, 821 bp) and corresponded to a total length of 4,544,250 bp, which is equivalent to 0.3% of the *P. equestris *genome (Table 1). The 5,535 BESs could be assembled into 340 contigs (average coverage = 2.99) and 4,518 singletons (data not shown). In terms of read-length distribution, 800-899 bp and 900-999 bp were the most abundant categories, accounting for 1,567 (28%) and 1,684 (30%) of all BESs, respectively (Figure 1). The GC content was 35.95%; this is comparable to the 34.09% previously estimated by buoyant density analysis of the genomic DNA of *P. amabilis *BLUME (*Sarcanthinae*; *Vandeae*) [29], indicating that the *Phalaenopsis *genome is AT-rich. Buoyant density analysis has also been used to study *Brassica maculate *R. BR. (*Oncidiinae*, *Vandeae*), *Cattleya schombocattleya *LINDL. (*Epidendrinae*, *Epidendrae*), and *Cymbidium pumilum *SWARTZ cv. "Gareth Latangor" (*Cymbidiinae*, *Vandeae*), which had GC contents of 32.05%, 34.09%, and 32.05%, respectively [29]. Thus, the available evidence suggests that most orchids have AT-rich genomes. All BES sequences generated herein have been deposited in GenBank under accession numbers HN176659-HN182163.

**Table 1:** Statistical analysis of Phalaenopsis equestris bacterial artificial chromosome (BAC) end sequences (BESs)

Total number of BESs	5,535
No. of paired BESs	5,360
No. of non-paired BESs	175
Total length (bp)	4,544,250
Minimum length (bp)	123
Maximum length (bp)	1,397
Average length (bp)	821
GC content	35.95%
Sequence composition	
Potential transposable elements (%)	1,272 (23.0)
Simple sequence repeats (%)	950 (17.2)
Protein coding regions (%)	641 (11.6)
Chloroplast sequences (%)	29 (0.5)
Unknown genomic sequences (%)	2,643 (47.7)

### Database sequence searches

The *P. equestris *BESs were subjected to sequence homology analysis using the RepBase and TIGR plant repeat databases, and RepeatMasker and BLAST were employed to predict repeat sequences and potential TEs, respectively. A total of 1,272 BESs (23.0% of total) were found to harbor putative TEs and repeats. The BESs were also RepeatMasked and compared to data in the NCBI non-redundant protein databases. A total of 641 (11.6%) were found to contain protein-coding sequences; of these, 29 BESs (0.5% of the total) contained putative chloroplast DNA-encoded genes (Table 1).

### Analysis of repetitive DNA in the BESs

The large genome size of *P. equestris *(1,600 kb) implies that the content of repetitive DNA could be high, rendering the genome more similar to that of maize than rice. The 29 BESs containing apparent chloroplast sequences were removed from analysis, and the remaining 5,506 BESs were screened for repetitive DNA sequences, using RepeatMasker and the TIGR plant repeat database. As for other eukaryotic genomes, that of *Phalaenopsis *was found to contain a significant proportion of repeat sequences and potential TEs; 1,272 BESs (23% of the total) contained such TEs (Table 1). This percentage was higher than that of apple (20.9%) [25], but lower than that of *Citrus clementina *(25.4%) [30], carrot (28.3%) [26], or *Musa acuminata *(36.6%) [24].

Among the 1,272 BESs containing potential TEs, more showed sequence homology to Class I retrotransposons (963 BESs, 75.7%) than to Class II DNA transposons (137 BESs, 10.8%), suggesting a ~7:1 Class I:Class II ratio in the genome (Table 2). The Class I retrotransposons could be further classified into Ty1/copia (163, 12.8%) and Ty3/gypsy (533, 41.9%) long-terminal-repeat (LTR) retrotransposons; LINE (95, 7.5%) and SINE (0, 0.0%) non-LTR retrotransposons; and other unclassified retrotransposons (172, 13.5%) (Table 2). Clearly, the LTR retrotransposons outnumbered those of the non-LTR form (696, 54.7% vs. 95, 7.5%); the number of unclassified retrotransposons (172, 13.5%). The next most abundant DNA repeat type was Class II DNA transposons, which included Ac/Ds (12, 0.9%), En/Spm (67, 5.3%), Mutator (15, 1.2%), Tourist, Harbinger, Helitron, Mariner (10, 0.8%), and other unclassified transposons (33, 2.6%). In total, 1,100 BESs were found to contain Class I retrotransposons and Class II DNA transposons. The other identified repeat sequences included miniature inverted-repeat transposable elements (MITEs; 2, 0.2%), centromere-related sequences (19, 1.5%), ribosomal RNA genes (33, 2.6%), and other unclassified repeat sequences (118, 9.3%). In total, such DNA was included in 172 BESs (13.4% of those harboring repetitive sequences) (Table 2).

**Table 2:** Number of bacterial artificial chromosome (BAC) end sequences (BESs) containing repetitive DNA

Class, subclass, group	No. of BESs	% of BESs with repetitive DNA
Class I retrotransposons	963	75.7
Ty1-copia	163	12.8
Ty3-gypsy	533	41.9
LINE	95	7.5
SINE	0	0.0
Unclassified retrotransposons	172	13.5
Class II DNA transposons	137	10.8
Ac/Ds	12	0.9
CACTA, En/Spm	67	5.3
Mutator (MULE)	15	1.2
Tourist/Harbinger/Helitron/Mariner	10	0.8
Unclassified Transposons	33	2.6
Miniature inverted-repeat transposable elements	2	0.2
Centromere	19	1.5
rRNA	33	2.6
Unclassified	118	9.3

Total	1272	100

### Functional annotation

**Figure 2:**
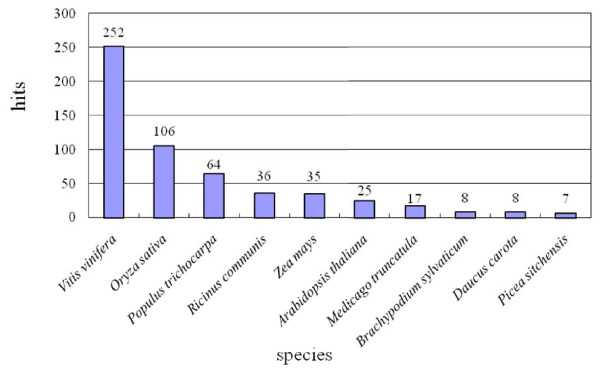
**Number of *P. equestris *BAC end sequences (BESs) that had significant hits in the NCBI database**. Among the protein matches, the top BLASTX match was to a protein of *V. vinifera*.

To identify protein-encoding regions, comparison of RepeatMasked BESs with the NCBI non-redundant protein databases revealed that 641 sequences (11.6%) contained apparent protein-encoding DNA (Table 1). Of these, 252 (39.3%) showed top BLAST matches search homologies to proteins from *V. vinifera*, whereas 106 (16.5%) best-matched proteins of *O. sativa *(Figure 2). This finding is consistent with BLAST data on orchid floral bud ESTs, which yielded top matches to *V. vinifera *followed by *O. sativa *[12,13]. At first glance, it seems very odd that orchid genes appear to be more highly related to a phylogenetically distant dicot species than to another monocot. Accumulation of additional orchid sequence data is needed to clarify this point.

BLASTN was used to compare the 641 BESs containing protein-encoding sequences to the sequences contained in our orchid EST databases [12,13]. We found that 417 BESs (65.1%) yielded matches and are known to be expressed in orchids (data not shown), whereas 224 (34.9%) did not show sequence matches when compared with the orchid EST databases.

Based on the fact that 641 predicted protein-encoding sequences covered 4,544 kb of the *Phalaenopsis *genome, as identified from 5,535 BESs, gene density analysis predicted that a gene should occur in every 7.1 kb of the *Phalaenopsis *genome. By comparison, banana (*M. acuminata*) has a gene density of 6.4 kb [24], rice (*O. sativa*) is predicted to have a gene every 6.2 kb [31], whereas *A. thaliana *is thought to have a gene every 4.5 kb [32].

**Figure 3:**
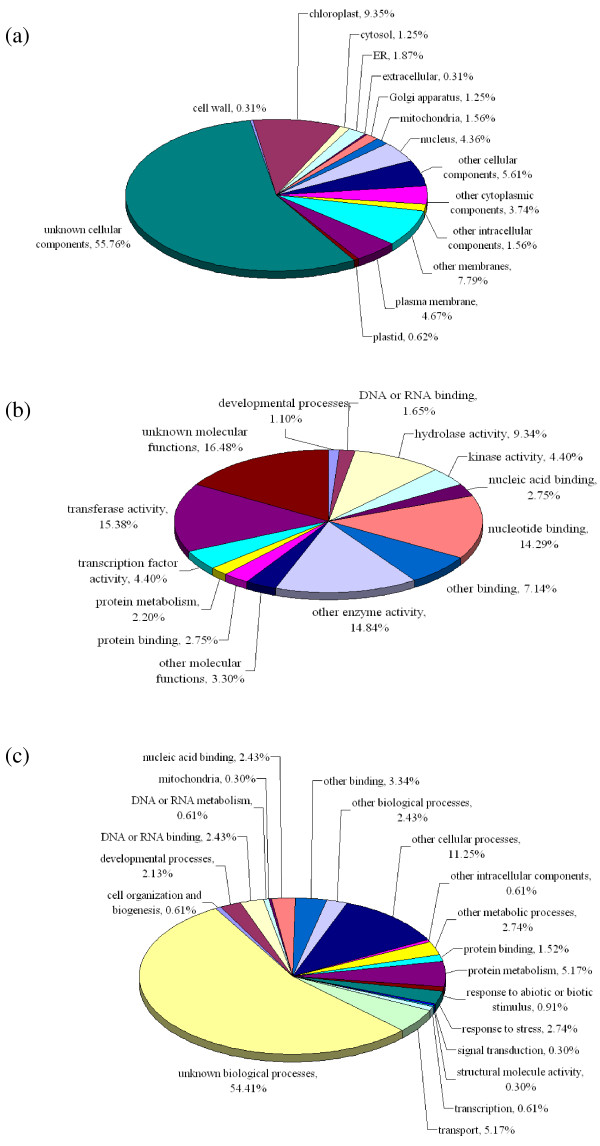
**Gene Ontology (GO) analysis of the *P. equestris *BESs into the categories of (a) cellular components, (b) molecular functions, and (c) biological processes**. Among the 641 BESs containing protein-encoding regions, 321 were annotated to the cellular components category, 182 to the molecular functions category, and 329 to the biological processes category.

The 641 BES-derived sequences showing homology to proteins in the NCBI non-redundant protein database were subjected to Gene Ontology (GO) annotation, and divided into three categories: cellular components (321 BESs), molecular functions (182 BESs), and biological processes (329 BESs). Among the 321 BESs in the cellular components category, 30 (9.35%) corresponded to chloroplast proteins, 25 (7.79%) to membrane proteins, and 18 (5.61%) to other cellular components. However, more than half of these BESs (179, 55.76%) encoded unknown cellular component proteins (Figure 3a). Sequences in the molecular functions category were distributed as follows: 16.48% (30 BESs) of unknown molecular function, 15.38% (28) with transferase activities, 14.84% (27) with other enzymatic activities, and 14.29% (26) with involvement in nucleotide binding (Figure 3b). Among the 329 BESs in the biological processes category, more than half corresponded to proteins involved in unknown biological processes (179 BESs, 54.41%), whereas the rest were associated with other cellular processes (37, 11.25%), protein metabolism (17, 5.17%), or transport functions (17, 5.17%) (Figure 3c).

BLASTX (E-values < 1e-5) was used to compare the RepeatMasked BESs to the protein databases of *O. sativa *(downloaded from the Rice Annotation Project Database, http://rapdb.dna.affrc.go.jp/download/index.html) and *V. vinifera *(downloaded from the NCBI *V. vinifera *protein database, ftp://ftp.ncbi.nih.gov/genomes/Vitis_vinifera/protein/). Of the 5,506 BESs, 550 (9.99%) were homologous to *V. vinifera *proteins. Thus, based on an estimated genome size of 1,600 Mb for *P. equestris*, it may be predicted that the total coding sequences of the *P. equestris *genome might represent approximately 159.8 Mb. If an average gene length of 3.4 kb, as in *V. vinifera *[33], is assumed, an estimate of the total gene content of the *P. equestris *genome is 47,007. When the rice genome was used for comparison, 504 *Phalaenopsis *BESs showed matches to the rice protein database, accounting for 9.15% of rice proteins. Similar estimations indicate that protein-encoding sequences cover 146.5 Mb of the *Phalaenopsis *genome and, assuming an average gene length of 2.7 kb in *Oryza *[34], predict that the *Phalaenopsis *genome contains 54,259 genes. These values are comparable to the 30,434 protein-encoding genes identified in the 487-Mb grape genome [33] and the 37,544 protein-encoding genes found in the 389-Mb rice genome [34]. Notably, gene distribution is fairly homogeneous along the chromosomes of rice and *Arabidopsis*, but genes are distributed more heterogeneously in *V. vinifera*. Pachytene karyotyping analyses of the *P. equestris *genome showed that the distribution of heterochromatin was pericentromeric, suggesting that genes of the *Phalaenopsis *orchids are more homogeneously distributed (personal communication, Dr. S. B. Chang, Department of Life Sciences, National Cheng Kung University, Taiwan). Based on the genome size of *Phalaenopsis*, we believe that both average gene length and gene distribution may be similar to those of the rice genome, and that approximately 54,259 heterogeneously distributed genes may be present.

### Simple sequence repeats (SSRs)

**Figure 4:**
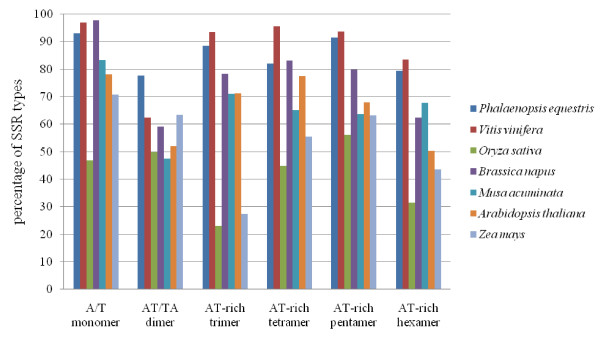
**Frequency of AT-rich repeat motifs in the nuclear genomes of *P. equestris *and selected other plant species**.

We identified 950 SSRs or microsatellites accounting for 17.2% of the obtained BESs (Table 1), and containing various repeat types (Table 3). BESs from *Arabidopsis thaliana*, *Brassica napus*, *M. acuminata*, *O. sativa*, *V. vinifera*, and *Zea mays *were downloaded and analyzed in parallel with those of *P. equestris*. Dinucleotide repeats, which are the most abundant repeat type in *M. acuminata *(47.7%), *B. napus *(36.2%), and *V. vinifera *(28.0%), were also the most common in the *P. equestris *genome, accounting for 34.37% of all SSRs. The next most common repeat type in *P. equestris *was mononucleotide in nature (29.9%) (Table 4). In addition, penta- and tri-nucleotide repeats accounted for 13.5% and 11.0%, respectively, of all SSRs in the *P. equestris *genome (Table 4). Among the mononucleotide repeats, far more A/T repeats (264 SSRs, 93%) than G/C repeats (20, 7%) (Table 3) were evident. Among the dinucleotide repeats, AT/TA was the most abundant (253, 77.6%), followed by AG/CT (47, 14.4%), AC/GT (24, 7.4%), and CG/GC (2, 0.6%) (Table 3). The average distance between SSRs was estimated to be 4.8 kb. Interestingly, this is the highest SSR frequency seen among plant genomes analyzed to date, including those of *Arabidopsis*, rapeseed (*B. napus*), banana (*M. acuminata*), rice, grape, and maize, which are estimated to have an average of 6.4, 9.2, 6.2, 9, 5.8, and 16.1 kb, respectively, between SSRs (Table 4). In *P. equestris*, grape, *B. napus*, and banana, dinucleotides were found to be the most abundant motifs, whereas trinucleotide SSRs predominated in rice and maize. *A. thaliana *was particularly rich in mononucleotide repeats. We noted some among-study variations in reported frequencies [24,26]; these appear to be mainly attributable to the use of different criteria for identifying SSRs. However, we can generally conclude that *P. equestris *and dicot plants such as grapevine, *B. napus*, and *A. thaliana *contain a high proportion of AT-rich motifs. The greatest repeat number (275) identified to date is found in the orchid genome (this study), whereas other monocots, such as *O. sativa *and *Z. mays*, appear to contain lower proportions of AT-rich motifs (Figure 4).

**Table 3:** Distribution of simple sequence repeats in P. equestris bacterial artificial chromosome (BAC) end sequences

Type	**No**.	Type	**No**.
A/T	264	AACTC/AGTTG	1
C/G	20	AACTT/AATTG	1
AC/GT	24	AAGAG/CTCTT	3
AG/CT	47	AAGGG/CCCTT	1
AT/AT	253	AAGTT/AATTC	1
CG/CG	2	AAGCC/CGGTT	1
AAC/GTT	22	AATAT/ATATT	4
AAG/CTT	12	AATCC/AGGTT	1
AAT/ATT	51	AATGC/ACGTT	1
ACC/GGT	3	ACACG/CTGTG	1
ACT/ATG	2	ACCTC/AGTGG	1
AGC/CGT	1	ACGAG/CTCTG	1
AGG/CCT	6	ACTAT/ATATG	1
AGT/ATC	5	AGAGG/CCTCT	4
CCG/CGG	2	AGGAT/ATCCT	1
AAAG/CTTT	2	AGGGC/CCCGT	1
AAAT/ATTT	20	AGGGG/CCCCT	1
AACT/ATTG	1	AAAAAG/CTTTTT	11
AATT/AATT	4	AAAAAT/ATTTTT	10
ACAG/CTGT	1	AAAACC/GGTTTT	15
ACAT/ATGT	12	AAAATT/AATTTT	1
ACCT/ATGG	4	AAAATC/AGTTTT	2
ACTC/AGTG	1	AAACCT/ATTTGG	1
AGAT/ATCT	2	AAACCC/GGGTTT	1
AGGG/CCCT	2	AAAGTC/AGTTTC	1
ACGT/ATGC	1	AAGGGC/CCCGTT	1
AAAAC/GTTTT	10	AACGTG/ACTTGC	4
AAAAG/CTTTT	20	AAGATC/AGTTCT	1
AAAAT/ATTTT	34	AATATT/AATTAT	1
AAACT/ATTTG	2	ACATAG/ATCTGT	1
AAAGT/ATTTC	2	ACATAT/ATATGT	2
AAAGG/CCTTT	1	ACCTGC/ACGTGG	2
AAATC/AGTTT	2	AACCCT/ATTGGG	1
AAATG/ACTTT	1	ACACCC/GGGTGT	1
AAATT/AATTT	30	ACCCCT/ATGGGG	1
AACAC/GTGTT	1	AGAGGC/CCGTCT	1

**Table 4:** Distribution and frequency of simple sequence repeats (SSRs) detected in different plant species

	***A. thaliana***	***B. napus***	***M. acuminata***	***O. sativa***	***P. equestris***	***V. vinifera***	***Z. mays***
No. of BESs	26,068	88,825	6,376	78,427	5,506	31,907	54,960
Total sequence length (bp)	13,987,589	39,551,595	4,517,901	69,423,321	4,520,220	18,117,956	37,410,959
Mononucleotides	40.3 (878)	7.5 (320)	0.8 (6)	9.1 (696)	29.9^a ^(284)^b^	6.0 (188)	7.2 (167)
Dinucleotides	13.1 (285)	36.2 (1,551)	47.7 (350)	19.9 (1,531)	34.3 (326)	28.0 (881)	15.4 (358)
Trinucleotides	21.2 (462)	20.4 (876)	20.6 (151)	28.9 (2,219)	11.0 (104)	18.7 (586)	35.7 (831)
Tetranucleotides	3.9 (84)	6.9 (294)	9.0 (66)	10.2 (783)	5.3 (50)	14.9 (467)	8.3 (193)
Pentanucleotides	15.3 (333)	21.2 (911)	13.1 (96)	21.4 (1,642)	13.5 (128)	21.4 (673)	21.6 (504)
Hexanucleotides	6.3 (137)	7.9 (338)	8.9 (65)	10.5 (804)	6.1 (58)	11.0 (347)	11.9 (276)
Total SSRs	2,179	4,290	734	7,675	950	3,142	2,329
SSR frequency^c^	6.4	9.2	6.2	9	4.8	5.8	16.1
Most frequent SSR motif	A/T	AT/TA	AT/TA	CCG/CGG	A/T	AT/TA	AGC/GCT

^a ^Percentage of SSRs in each category

^b ^Number of SSRs in each category

^c ^Average estimated distance between SSRs (kb)

SSR markers have been widely used for genotyping of crop plant species [35,36]. Of the 950 detected SSRs, we chose 206 for use in primer design (Additional file 1), and subsequently assessed whether the primers could successfully distinguish 12 *Phalaenopsis *species, based on allelic polymorphisms. More than 85% of primer pairs successfully amplified products from at least 1 of the 12 tested *Phalaenopsis *species, all of which have been extensively used as parents in breeding programs (Additional file 2). The cross-species transferability rate of the tested SSRs ranged from 76.1- 54.8% (Additional file 2), and most primer pairs produced polymorphic bands in the majority of tested *Phalaenopsis *species (Additional file 3). In a future study, we will examine the efficacy of such SSR markers for genotyping of commercial orchid cultivars.

### Comparative mapping of orchid BAC ends to other plant genomes for identification of microsynteny

To examine syntenic relationships between orchid and other plant species, orchid BESs were BLAST-searched against the whole-genome sequences of *A. thaliana*, rice (*O. sativa*), poplar (*Populus trichocarpa*), and grape (*V. vinifera*). BAC end pairs of appropriate orientation and no more than 50-300 apart on any given chromosome were considered to be potentially collinear with the target genome. Our results revealed that 142 *Phalaenopsis *BESs, including 14 end-pair sequences, yielded hits in the poplar genome. Twelve pairings mapped together on various chromosomes, but only 1 such pair was found within 50-300 kb of another, suggesting colinearity (Table 5). The next greatest number of hits was obtained when the grape genome was compared with that of our BESs; significant hits were obtained for 123 BESs, including 12 BAC end-pairs, 10 of which mapped together on various chromosomes. One of the 10 BAC end-pairs was found on different contigs of the same chromosome; presumably these contigs are mutually close, maybe even within 50-300 kb (Table 5). Ninety-four orchid BESs, including 12 paired ends, showed significant hits to the *Arabidopsis *genome. Eleven of the BAC end-pairs colocalized on various chromosomes, but none were less than 50-300 kb apart (Table 5). The mapping of orchid BESs to the genome of rice produced 83 BES hits, including six paired ends that colocalized on various chromosomes, but were not within 50-300 kb of each other (Table 5).

**Table 5:** Microsynteny between Phalaenopsis and A. thaliana, O. sativa, P. trichocarpa and V. vinifera

	***A. thaliana***	***O. sativa***	***P. trichocarpa***	***V. vinifera***
No. of hits	94	83	142	123
Pair ends	12	6	14	12
Same chromosome	11	6	12	10
50- to 300-kb sequence	0	0	1	1^a^

^a ^The whole-genome sequence data of *V. vinifera *shows that each individual chromosome contains several contigs. Thus, we were unable to determine the exact distances between the BAC pair ends that mapped together on the various chromosomes of the grape genome.

The simple Monte Carlo Test [37] was used to assess the statistical significance of the microsynteny results. The sequences of each *Phalaenopsis *BES were randomly shuffled 100 times to obtain 550,600 simulated sequences, which were next BLASTN- compared to the genomic sequences of poplar, grape, rice, and *Arabidopsis*. None of the simulated sequences mapped to the genomes of the various plants, suggesting that our results with respect to microsynteny mapping of orchid BESs onto other plant genomes are meaningful.

Most paired ends that mapped together on plant chromosomes were annotated as ribosomal DNA (rDNA); these sequences accounted for 10 of 14 end-pairs in poplar, 10 of 12 in grape and *Arabidopsis*, and all 6 end-pairs of rice. In addition, all end-pairs that contained rDNA mapped to a single chromosome in each plant species.

Twenty-nine orchid BESs containing *Phalaenopsis *chloroplast genome sequences showed matches with genomic sequences of the four plant genomes: 25 BESs with the grape genome, 24 with the rice genome, 21 with the poplar genome, and 2 with the *Arabidopsis *genome (Table 6; assessed using BLASTN with an E-value <1e-30). Moreover, such BESs also showed matches within chloroplast DNA sequences from the four plant genomes: 28 with the grape genome, 24 with rice, 27 with poplar, and 25 with *Arabidopsis *(E-value <1e-30). Transfer of chloroplast DNA to the nucleus is well-known to result in insertion of chloroplast DNA into nuclear chromosomes. In rice, 421-453 chloroplast insertions have been identified throughout the 12 chromosomes, forming 0.18-0.19% of the rice nuclear genome [34]. In the present work, the 29 orchid BESs containing *Phalaenopsis *chloroplast DNA included 12 paired and 5 unpaired BES end sequences. This suggests that at least some of these BESs may be located in the *Phalaenopsis *nuclear genome, rather than representing experimental contamination with chloroplast DNA.

**Table 6:** Number of bacterial artificial chromosome (BAC) end sequences (BESs) containing Phalaenopsis chloroplast DNA with hits to the nuclear and chloroplast DNA of A. thaliana, O. sativa, P. trichocarpa and V. vinifera

	***A. thaliana***	***O. sativa***	***P. trichocarpa***	***V. vinifera***
Nuclear DNA	2	24	21	25
Chloroplast DNA	25	24	27	28

Previous reports found negligible colinearity between onion (Asparagales) and rice (Poales) [38,39]. The Asparagales include a number of economically important plants, such as asparagus, chives, garlic, leeks, onions, and orchids. Similarly, the well-documented high-level synteny among grass genomes is not found among members of other monocot orders [*e.g., Musa *(Zingiberales)], even though microsynteny persisted beyond the time of divergence of the Commelinid orders Poales and Zingiberales [40]. In the present work, we failed to find any syntenic relationship between orchid and rice sequences, confirming the previously noted lack of synteny between Asparagales and Poales.

Whole-genome duplication, resulting in polyploidy, occurred in early monocots such as the Poales and Zingiberales [40,41]. Duplication, and gene loss and rearrangements occurring after such whole-genome duplication, led to subsequent evolution and increases in morphological complexity. This may also have occurred in the orchid genome, as suggested by the presence of the four *AP3*-like paralogs that form the basis for the complicated floral morphologies of *Phalaenopsis *[16]. Interestingly, these paralogs are present in at least four out of the five subfamilies of the Orchidaceae [42]. Future whole-genome sequencing of *Phalaenopsis *should provide additional insights into genome reorganization and help to clarify differences in genomic composition between orchids and other plant species.

## Conclusions

This analysis of *Phalaenopsis *BAC end sequences offers the first insights into the composition of the *Phalaenopsis *genome in terms of GC content, transposable elements present, protein-encoding regions, SSRs, and potential microsynteny between *Phalaenopsis *and other plant species. The protein sequence similarities between *Phalaenopsis *and grape and the potential microsynteny between *Phalaenopsis *and poplar are interesting and should be confirmed by large-scale BAC end sequencing. The present work also provides a good basis for additional sequence analysis of *Phalaenopsis *BAC libraries, and will also encourage contig fingerprinting and physical mapping of the *Phalaenopsis *genome.

## Methods

### Orchid BAC-end sequencing

BAC clones were randomly chosen from 96-well microplates and inoculated into 96-well deep-well plates containing 1.5 ml of 2x LB medium with 12.5 μg/ml chloramphenicol. Plates were incubated at 37°C with continuous shaking at 100 rpm for 20-24 h. BAC-end sequencing was performed using BigDye^® ^Terminator v 1.1 and ABI PRISM^® ^3730 DNA Analyzer technologies (Applied Biosystems, Life Technologies Corporation, Foster City, CA). The work was performed by the Sequencing Core Facility of the National Yang Ming University Genome Center (YMGC, Taipei, Taiwan) and the Arizona Genomics Institute DNA Sequencing Center (AGI, Tucson, AZ).

BESs were base-called and processed using CodonCode Aligner software (Version 2.0; CodonCode Corporation, Dedham, MA), which integrates the PHRED program [43]. We used the default parameters of "maximize region with error rate < 0.1" to trim bases from flanking sequences and next performed the operations "move all sequences shorter than 25 bases to trash" and "move all sequences with fewer than 50 Phred-20 bases to trash." Vector sequences were trimmed using Sequencher V4.1 (Gene Codes Corporation, Ann Arbor, MI) with reference to the pIndigoBAC5 DNA sequence. We next discarded all BESs < 100 bp, which yielded a set of 5,535 BESs. These were BLASTN-searched against the chloroplast DNA sequence of *P. aphrodite *subsp. *formosana *(GenBank accession no. NC_007499) [44] and the mitochondrial DNA sequence of *O. sativa *(*japonica*) cultivar Nipponbare (accession no. DQ167400) [45], using a stringent threshold of < 1e-50.

### Analysis of repetitive sequences

BESs were analyzed for repetitive sequences using RepeatMasker [46]. We applied the same default conditions as were employed in construction of the *Arabidopsis*, rice, and maize sections of the RepBase Update databases [47]. We next used BLASTN and TBLASTX to search the TIGR plant repeat database (downloaded on Sep. 5, 2009) [48] with E-values < 1e-10 and < 1e-5, respectively. Repetitive sequences were annotated using the RepeatMasker default setting or were classified employing the TIGR codes for repetitive plant sequences.

### Functional annotation of *Phalaenopsis *BESs

The 5,506 RepeatMasked BESs were further analyzed for protein-encoding regions via BLASTX searching of NCBI non-redundant protein databases (E-values < 1e-5). Protein-encoding BESs were BLASTN-searched against our in-house orchid EST databases [12,13], using an E-value < 1e-20. BESs containing protein-encoding regions homologous to proteins found in the NCBI non-redundant protein databases were further analyzed in terms of Gene Ontology (GO) annotations, using a BLASTX search of the *Arabidopsis *genome annotation database (ftp://ftp.arabidopsis.org/home/tair/Genes/TAIR9_genome_release/; E-value < 1e-7). Categories were assigned based on biological, functional, and molecular annotations available from GO http://www.geneontology.org/.

### SSR identification and marker development

BESs from *A. thaliana*, *B. napus*, *M. acuminata*, *O. sativa*, *V. vinifera*, and *Z. mays *were obtained from the Genome Survey Sequences (GSS) database of the NCBI (downloaded on Dec. 11, 2009), and subjected to SSR analysis using the same parameters as were employed in our analysis of orchid BESs. SSR types (mononucleotide to hexanucleotide) were identified using the MIcroSAtellite (MISA) tool [49]; the analysis required a minimum length of 20 bases for mononucleotide repeats and at least 15 bases for dinucleotide-to-hexanucleotide repeats, and allowed a maximum of an 100-nt interruption if compound repeats were encountered. Primer3 software http://frodo.wi.mit.edu/primer3/ was used for primer design. The following parameters were employed: (1) BESs with a minimum of eight dinucleotide, five trinucleotide, four tetranucleotide, three pentanucleotide, or three hexanucleotide repeats; or an SSR motif length longer than 15 bp; (2) primer lengths of 18-25 nt, with 20 nt being considered optimal; and (3) predicted PCR products of 150-350 bp. A total of 206 primer pairs (Additional file 1) were synthesized and used to amplify genomic DNA from 12 *Phalaenopsis *species: *P. amabilis, P. aphrodite *subsp. *formosana, P. schilleriana, P. stuartiana, P. equestris, P. sanderiana, P. lueddemanniana, P. amboinensis, P. pulcherrima, P. fasciata, P. venosa*, and *P. gigantea*. Genomic DNA was isolated from leaf samples using a BioKit Plant Genomic DNA Purification Kit. PCR was performed using 10 ng of genomic DNA, paired primers, dNTPs, 10× buffer, and *Taq *polymerase, in 20-μl reaction volumes. The PCR amplification conditions were: 94°C for 5 min followed by 45 cycles of 94°C for 60 sec, annealing (45-60°C) for 40 sec and 72°C for 40 sec, and a final extension for 5 min at 72°C. PCR products were separated on either 3% (w/v) agarose or 8% (w/v) denaturing polyacrylamide gels, which were next stained with ethidium bromide for visualization of SSR bands.

### Microsynteny between *P. equestris *and *A. thaliana*, *O. sativa*, *P. trichocarpa*, and *V. vinifera*

*Phalaenopsis *BESs (not RepeatMasked) were compared with the genomic sequences of *A. thaliana*, *O. sativa*, *P. trichocarpa*, and *V. vinifera *(downloaded from the NCBI database ftp://ftp.ncbi.nih.gov/genomes/ on Feb. 27, 2010) by means of a BLASTN search with an E-value < 1e-10. To identify BACs from the *Phalaenopsis *library that showed microsynteny with the reference genomes, as described in a previous study on *Musa *[24], we searched the *Phalaenopsis *genomic sequence for BESs, both ends of which showed highly significant matches to *A. thaliana*, *O. sativa*, *P. trichocarpa*, or *V. vinifera *sequences, and that were located 50-300 kb apart in the *Phalaenopsis *genome.

The simple Monte Carlo Test [37] was used to assess the statistical significance of microsynteny between *Phalaenopsis *BESs and the genomes of *A. thaliana*, *O. sativa*, *P. trichocarpa*, and *V. vinifera*. The sequence of each *Phalaenopsis *BES was randomly shuffled 100 times to obtain 550,600 simulated sequences, which were then compared, using BLASTN, to the *Arabidopsis*, rice, poplar, and grape genomes (E-value < 1e-10).

## List of abbreviations

BAC: bacterial artificial chromosome; BESs: BAC end sequences; GO: Gene Ontology; MISA: MIcroSAtellite identification tool; SSRs: simple sequence repeats;

## Authors' contributions

CCH performed the bioinformatic analyses for genes, transposable elements, and microsynteny. YLC, YLL, and YTK performed the SSR prediction and verification. TCC and WLW constructed the *Phalaenopsis *BAC library for sequencing. WCT, YYH, YWC, WLW and HHC participated in the study design and data analysis. CCH, YLC, YLL, WLW, and HHC drafted the manuscript. All authors read and approved the final manuscript.

## Supplementary Material

Additional file 1 **List of 206 SSR markers developed from the *P. equestris *BAC end sequences**. The data provided represent details of the SSR markers *e.g*. marker name, accession number, primer sequence, PCR condition, expected product size and indication of whether primer pairs successfully amplified at least one of the 12 tested *Phalaenopsis *species. Click here for file

Additional file 2 **Rate of successful amplification of *P. equestris *SSRs among 12 *Phalaenopsis *species**. The cross-species amplification rate of the 206 SSR markers was assessed in 12 *Phalaenopsis *species. Click here for file

Additional file 3 **PCR amplification profiles of four *P. equestris* SSR markers in 12 *Phalaenopsis* species.** 12 *Phalaenopsis* species listed in Additional file 2 were used for polymorphism analysis of four SSR markers PeGBMS114, PeGBMS117, PeGBMS126 and PeGBMS216 listed in Additional file 1. Click here for file
