# Supplementary material for: An overview of the Phalaenopsis orchid genome through BAC end sequence analysis
Source: BMC Plant Biol. 2011 Jan 6;11:3. doi: 10.1186/1471-2229-11-3 (PMC3027094; doi:10.1186/1471-2229-11-3)
Supplement: Additional file 3 — PCR amplification profiles of four P. equestris SSR markers in 12 Phalaenopsis species. 12 Phalaenopsis species listed in Additional file 2 were used for polymorphism analysis of four SSR markers PeGBMS114, PeGBMS117, PeGBMS126 and PeGBMS216 listed in Additional file 1. [file 1471-2229-11-3-S3.DOC]

**
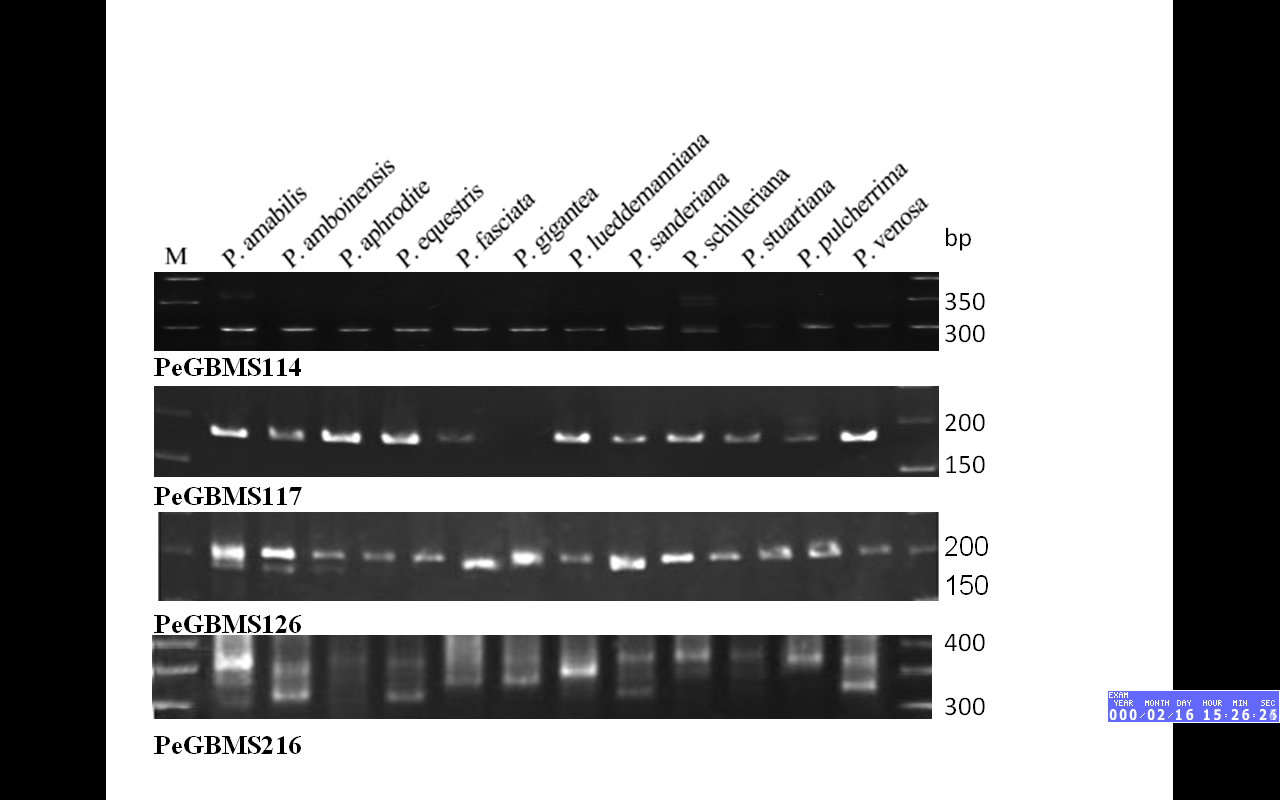
**

**Additional file 3:** PCR amplification profiles of *P. equestris* microsatellite markers in 12 *Phalaenopsis* species. Amplicons were resolved in 8% PAGE. M: 50 bp DNA ladder.
